# Implant selection and causes of aseptic failure in conversion from unicompartmental to total knee arthroplasty: A high‐volume centre series

**DOI:** 10.1002/jeo2.70680

**Published:** 2026-02-28

**Authors:** Julius Brendler, Lorenzo Impieri, Christoph Linhart, Michael Najfeld, Robert Hube, Daniel Pfeufer

**Affiliations:** ^1^ Department of Orthopedics and Trauma Surgery, Musculoskeletal University Center Munich (MUM) University of Munich (LMU) Munich Germany; ^2^ Orthopädische Chirurgie München (OCM) Munich Germany; ^3^ Residency Program in Orthopedics and Traumatology University of Milan Milan Italy

**Keywords:** knee osteoarthritis, UKA complications, UKA revisions, UKA to TKA, unicompartimental knee arthroplasty

## Abstract

**Purpose:**

This study aimed to describe the causes of aseptic failure leading to conversion from unicompartmental knee arthroplasty (UKA) to total knee arthroplasty (TKA) and to report implant selection and mid‐term outcomes in a high‐volume orthopaedic centre.

**Methods:**

In this retrospective single‐centre case series, 27 patients (13 women, 14 men; mean age 68.9 years, range 55–81) undergoing conversion from UKA to TKA between January 2013 and December 2020 were included. Causes of UKA failure, type of revision implant (posterior‐stabilised [PS], constrained posterior‐stabilised [CPS] or legacy constrained condylar knee [LCCK]), and use of tibial stem extensions were recorded. Implant survival after conversion was estimated using Kaplan–Meier analysis, and re‐revision rates were documented. Absolute numbers with percentages were reported.

**Results:**

The most common causes of UKA failure were periprosthetic tibial fracture (10/27; 37%), tibial component loosening (8/27; 30%) and progression of osteoarthritis (7/27; 26%). PS implants were used in 17 patients (63%; 6 with cemented tibial stem), CPS in 9 (33%; all with stems) and LCCK in 1 (4%; with stem). Overall, tibial stem extensions were used in 16 cases (59%). At a mean follow‐up of 23 months (range 0–89), 2 patients (7%) underwent re‐revision: one for tibial loosening and one for instability. Kaplan–Meier analysis estimated a median implant survival of 74 months (SD 17.1; 95% CI: 40.6–107.4 months).

**Conclusion:**

In this specialised high‐volume centre, conversion of UKA to TKA was most performed for mechanical failure or disease progression. Most cases could be managed with PS implants, with selective use of stems or higher constraint when indicated. Re‐revisions were rare, but findings should be interpreted cautiously given the small sample size, variable follow‐up and potential selection bias.

**Level of Evidence:**

Level IV.

**Abbreviations**
BMI: body mass index
CCK: condylar constrained knee
CPS: constrained posterior‐stabilised
OA: osteoarthritis
PS: posterior‐stabilised
TKA: total knee arthroplasty
UKA: unicondylar knee arthroplasty

## INTRODUCTION

Unicompartmental knee arthroplasty (UKA) is an effective treatment for isolated medial or lateral compartment osteoarthritis, offering faster recovery, lower perioperative morbidity and better functional outcomes compared to total knee arthroplasty (TKA) in selected patients [4]. However, revision rates following UKA remain higher than those reported for primary TKA, with registry data indicating cumulative revision rates at 10 years ranging from 10% to 15% [8]. Understanding the causes of UKA failure and subsequent revision to TKA is crucial to improve patient selection, surgical technique and implant design.

The most common causes of UKA revision include aseptic loosening, progression of osteoarthritis in the nonreplaced compartments, polyethylene wear, instability and infection [13, 15].

These failures often necessitate conversion to TKA, which can be a technically demanding procedure due to issues such as bone loss, soft tissue imbalance and the presence of retained cement or hardware from the primary UKA [16].

Studies suggest that most UKA revisions can be successfully managed with primary TKA implants, although a subset of patients may require the use of augments, stems or constrained inserts to address instability or bone deficiency [2, 24].

A recent study on 35 UKA‐to‐TKA revision showed that 40% of revision implants were posterior stabilised revision TKA, followed by 34% of condylar constrained designs and 23% of rotating hinged TKA. Only one patient was revised to a cruciate retaining primary implant (3%) [5]. Another study performed in 2012 involving 33 UKA cases revised due to aseptic issues reported that 55% of these revisions could not be managed with a standard TKA and required more complex implants, including the use of stems or augments [7]. These procedures present additional surgical challenges and carry a higher risk of further complications, but they may be necessary to reduce the likelihood of recurrent tibial loosening and early repeat revision. Supporting this approach, a registry analysis of over 4000 revised UKAs demonstrated improved re‐revision‐free survival when tibial stem extensions were utilised, either alone or alongside augments [11].

Evidence indicates that outcomes of UKA‐to‐TKA revisions, while generally satisfactory, may not fully match those of primary TKA and can have a major risk of re‐revision, which is similar to the risk of re‐revision of a primary TKA, underscoring the need for careful preoperative planning and appropriate implant selection [10, 12].

The present study aims to analyse the causes of UKA‐to‐TKA revision in our institution, focusing on implant selection during revision surgery, and to evaluate the re‐revision rate of the TKA. Given that the etiology of UKA failure can influence the technical challenges and implant selection during conversion to TKA, we aim to describe these causes alongside the intraoperative decision‐making for implant choice. Our hypothesis is that in a high‐volume centre, most UKA failures can be managed with posterior‐stabilised (PS) implants under careful intraoperative assessment, with selective use of stems or higher constraint when indicated.

## METHODS

### Ethical approval

Ethical approval for this study was obtained from the local ethics committee (Ethik‐Kommission der Bayerischen Landesärztekammer, Mühlbauerstraße 16, 81677 München; approval no. 24075). As this study retrospectively analysed routine patient data, no individual informed consent was required.

### Study design and patient population

This retrospective single‐centre study included all patients who underwent conversion from UKA to TKA for aseptic failure between 1 January 2013 and 31 December 2020, at a specialised orthopaedic clinic (Orthopädische Chirurgie München [OCM]) in Munich, Germany. Four senior surgeons, each performing > 300 arthroplasties annually, performed all procedures. Patients were identified using the German OPS code 5‐823.1b (conversion of a cemented UKA to a cemented TKA).

The cohort comprised 27 patients (13 women, 14 men), including 18 medial‐fixed bearing ZUK (Lima Corporate®) and 9 PPK (Zimmer Biomet®) implants. Early failures (< 5 years after index surgery) occurred in 21 patients, and late failures (≥ 5 years) in 6 patients. The mean age at conversion was 68.9 years (median 69; interquartile ranges [IQR] 64–74), and median body mass index (BMI) was 29.3 kg/m². Mean follow‐up after conversion was 23 months (median 13; range 0–89 months). Mean time from primary UKA to TKA was 34.9 months (median 14; range 0–180 months). The reported follow‐up range includes one patient with 0 months follow‐up due to early conversion from UKA to TKA.

### Definitions and endpoints

Aseptic failure was defined as any revision surgery involving implant exchange and served as the primary endpoint. Re‐revision of the TKA was defined as the secondary endpoint. Implant loosening was diagnosed based on combined clinical assessment and radiographic evaluation according to Knee Society criteria using three standardised radiographic projections.

### Surgical procedure

All revision surgeries were performed with a standard medial parapatellar arthrotomy. Prosthetic components were explanted, and extensive debridement was performed to remove fibrous tissue. Implant selection was determined intraoperatively by the attending surgeon, based on patient‐specific factors including age, bone stock quality, joint stability, extent of bone loss and indication for revision. In this cohort, most conversions were managed using PS implants. In our high‐volume centre, these implants could be safely used when intraoperative findings, including bone quality and knee stability, allowed.

- Minor defects with mechanically stable knees were treated with PS primary TKA components (Zimmer Biomet®).
- Constrained posterior‐stabilised (CPS) components with optional cemented tibial stem extensions were used in cases with ligamentous laxity or additional fixation requirements.
- Severe instability or extensive bone loss was managed with legacy condylar constrained knee (LCCK) implants (Zimmer Biomet®).

Cemented tibial stems were used routinely with CPS and LCCK implants and selectively for PS implants depending on bone quality. This intraoperative decision‐making introduces potential selection bias, which should be considered when interpreting implant‐specific outcomes. Given the heterogeneity in patient characteristics and indications for revision, outcomes for PS, CPS or constrained condylar knee (CCK) implants are reported descriptively only. The use of PS implants should be interpreted cautiously, generally reserved for cases where intraoperative assessment indicates sufficient bone quality and knee stability.

### Statistical analysis

Data were collected using Microsoft Excel® and analysed with IBM SPSS Statistics Version 29®. Descriptive statistics were used to summarise the cohort. Means and ranges were calculated for parametric variables, and medians with IQR for nonparametric variables. Implant survival was assessed using Kaplan–Meier analysis. Numbers at risk were recorded for each time point, and median survival with standard deviation and 95% confidence intervals were reported.

## RESULTS

### Patient cohort

A total of 27 patients (13 women, 14 men) underwent UKA‐to‐TKA conversion for aseptic failure between January 2013 and December 2020. The mean age at conversion was 68.9 years (median 69; IQR: 64–74), and the median BMI was 29.3 kg/m². Early failures (< 5 years after UKA) occurred in 21 patients, and late failures (≥ 5 years) in 6 patients. Pooling these early and late failures may introduce heterogeneity in surgical complexity and implant selection. Therefore, interpretations of implant‐specific outcomes, including the use of PS designs, should be considered descriptive. The mean follow‐up after conversion was 23 months (median 13; range 0–89 months). The mean survival time of the UKA before conversion was 34.9 months (median 14; range 0–180 months). The patient demographics are summarised in Table 1.

**Table 1:** Patient demographics.

Variable	Value
Number of patients	27
Sex (female: male)	13: 14
Mean age at primary UKA surgery, years	68.9
Median; IQR (Q1–Q3)	69; 11 (64–75)
Mean BMI, kg/m^2^	29.33
Median; IQR (Q1–Q3)	29.3; 4.9 (26.65–31.55)
Mean follow‐up, months	23
Median; IQR (Q1–Q3)	13; 29 (5–34)
Mean time from UKA to TKA, months	34.67
Median; IQR (Q1–Q3)	14; 52 (3–55)

Abbreviations: BMI, body mass index; IQR, interquartile range; TKA, total knee arthroplasty; UKA, unicompartmental knee arthroplasty.

### Indications for conversion

**Figure 1:**
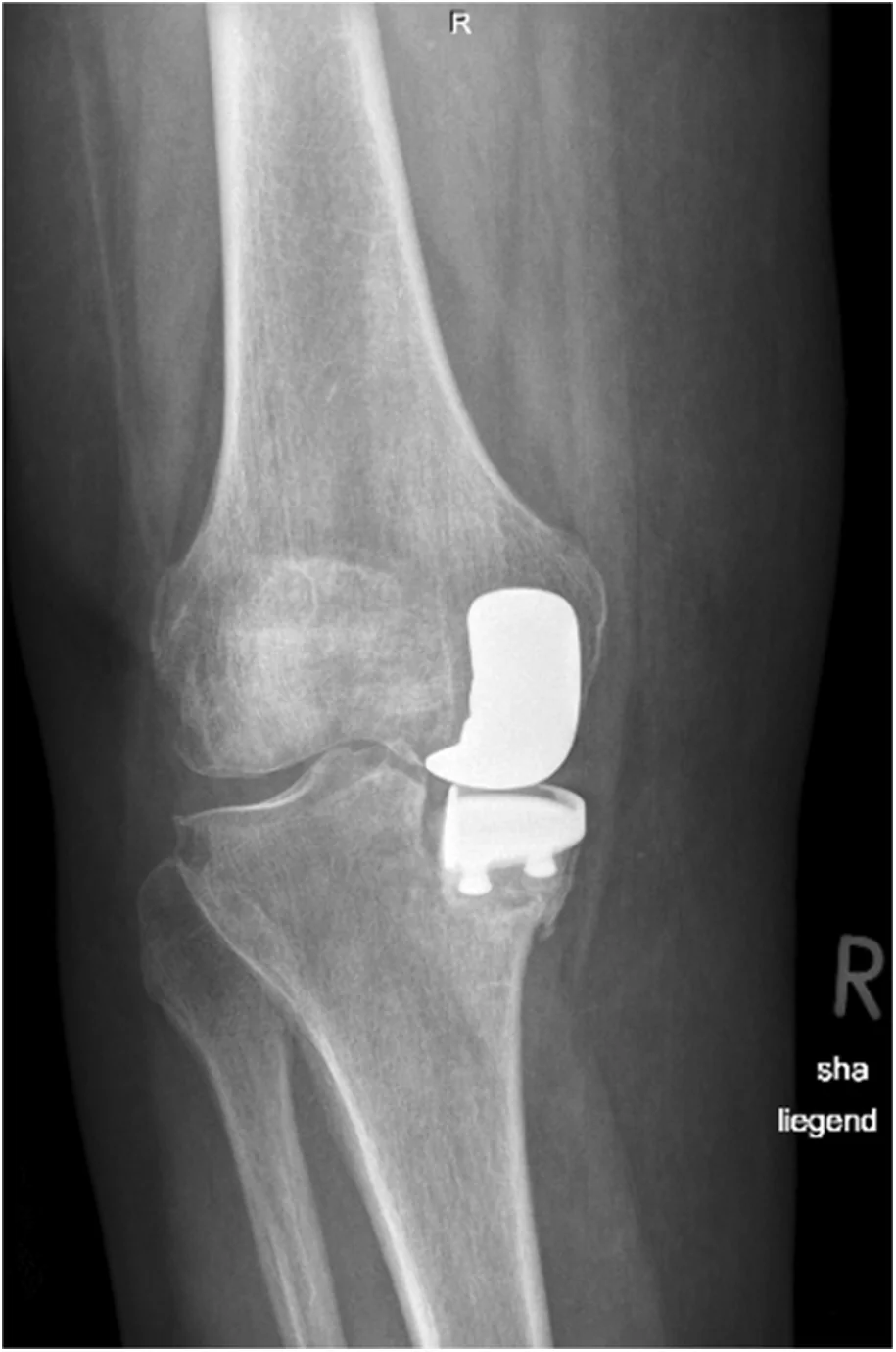
PPF after UKA (a.p.). PPF, periprosthetic fracture; UKA, unicompartmental knee arthroplasty.

**Figure 2:**
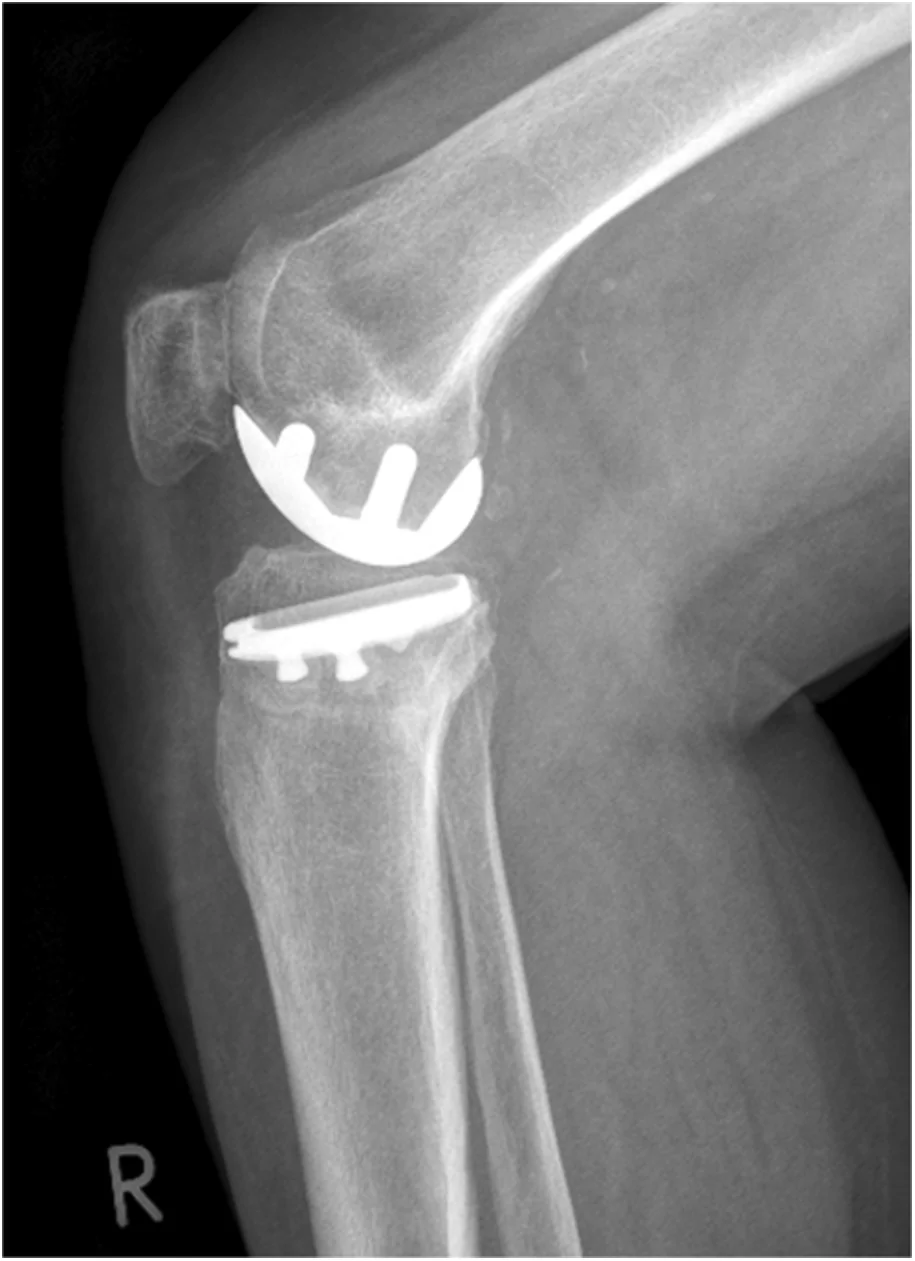
PPF after UKA (lateral). PPF, periprosthetic fracture; UKA, unicompartmental knee arthroplasty.

**Figure 3:**
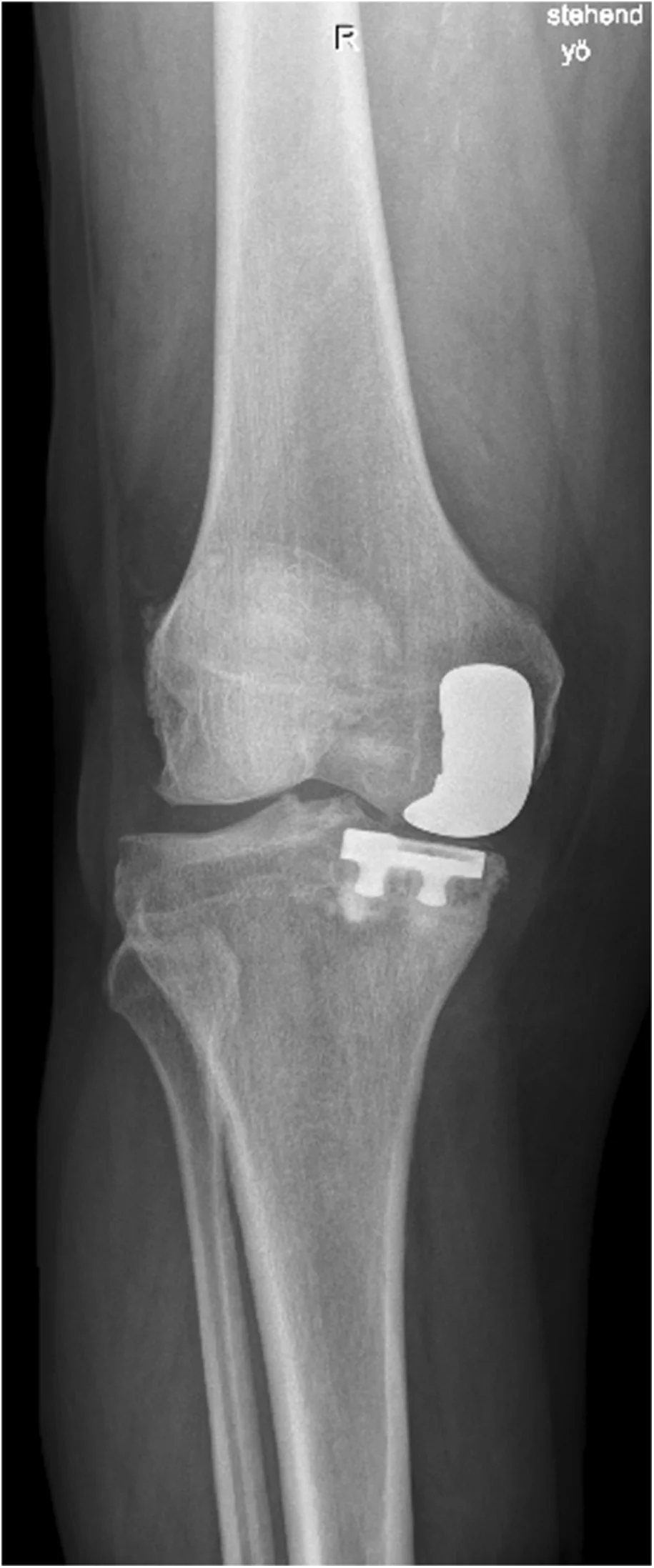
Aseptic tibial component loosening after UKA (a.p.). UKA, unicompartmental knee arthroplasty.

**Figure 4:**
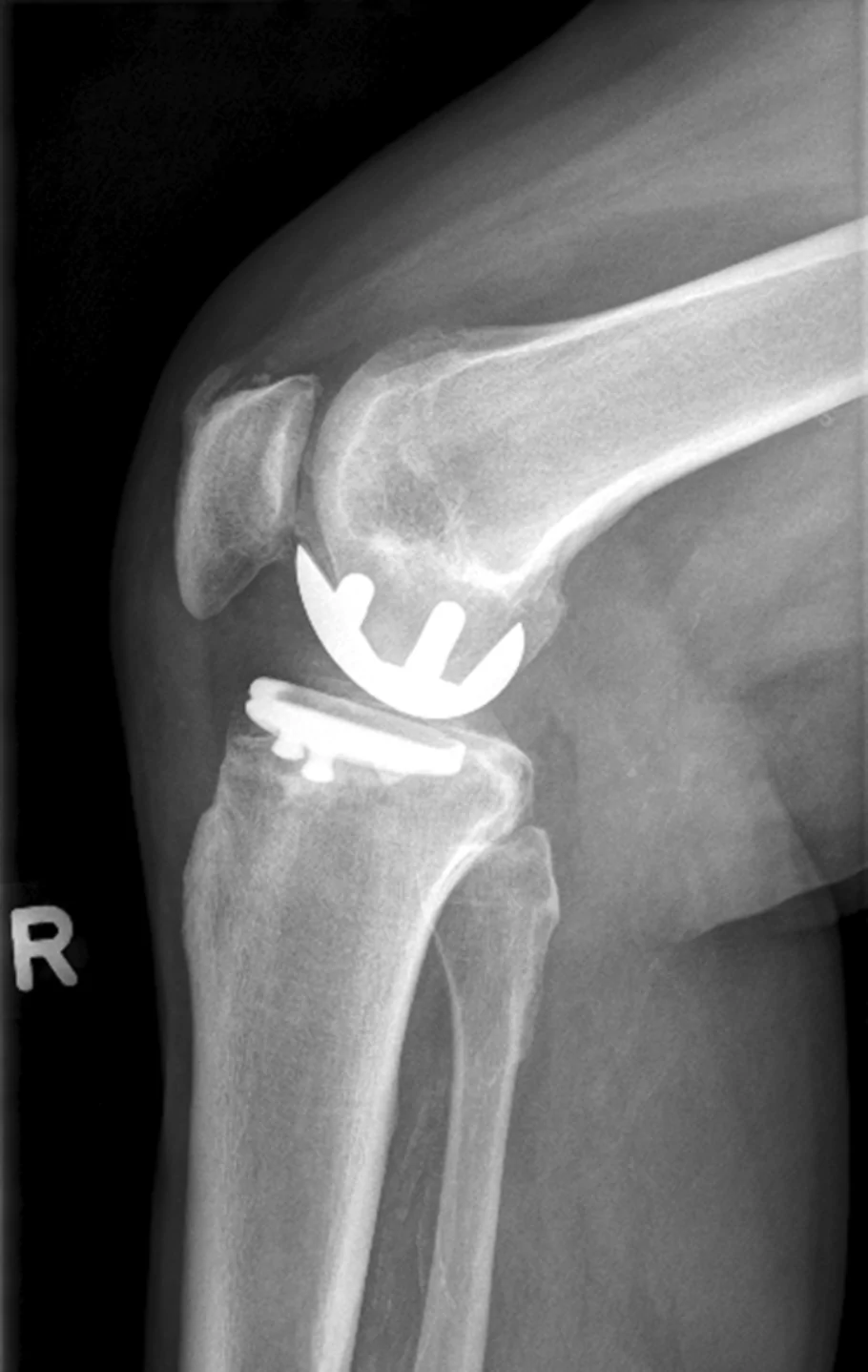
Aseptic tibial component loosening after UKA (lateral). UKA, unicompartmental knee arthroplasty.

**Figure 5:**
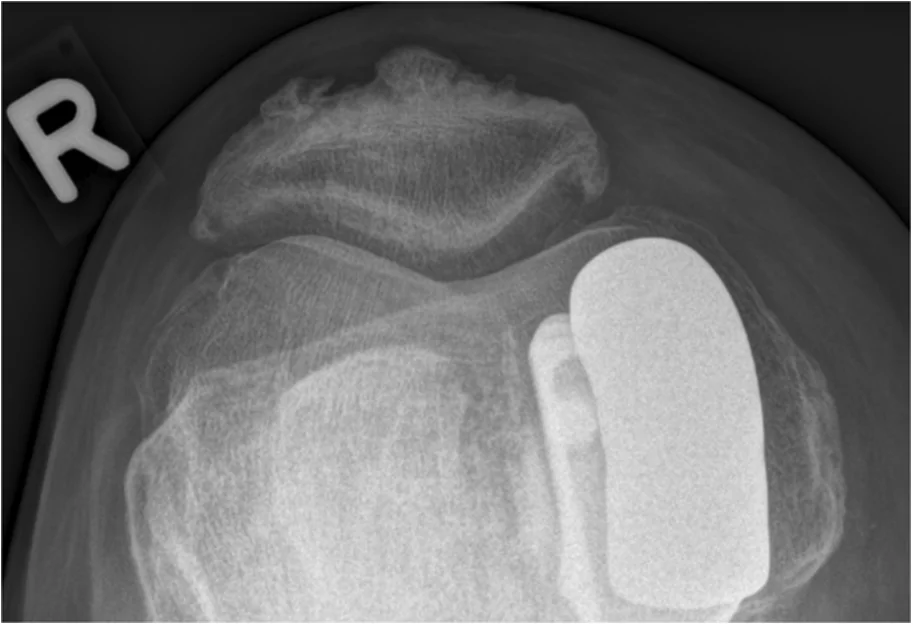
Aseptic tibial component loosening after UKA (patello‐femoral). UKA, unicompartmental knee arthroplasty.

The most common causes of UKA failure were periprosthetic tibial fracture (*n* = 10; 37%) (Figures 1 and 2), tibial component loosening (*n* = 8; 30%) (Figures 3, 4, 5), and progression of osteoarthritis (*n* = 7; 26%). One patient (4%) was revised for persistent pain without an identifiable mechanical cause, and one patient (4%) for instability. The indications for conversion are listed in Table 2.

**Table 2:** Indications for UKA‐to‐TKA conversion.

Cause of failure	*n* (%)
Periprosthetic tibial fracture	10 (37)
Tibial component loosening	8 (30)
OA progression	7 (26)
Persistent pain	1 (4)
Instability	1 (4)

Abbreviations: OA, osteoarthritis; TKA, total knee arthroplasty; UKA, unicompartmental knee arthroplasty.

### Implant choice and fixation

**Figure 6:**
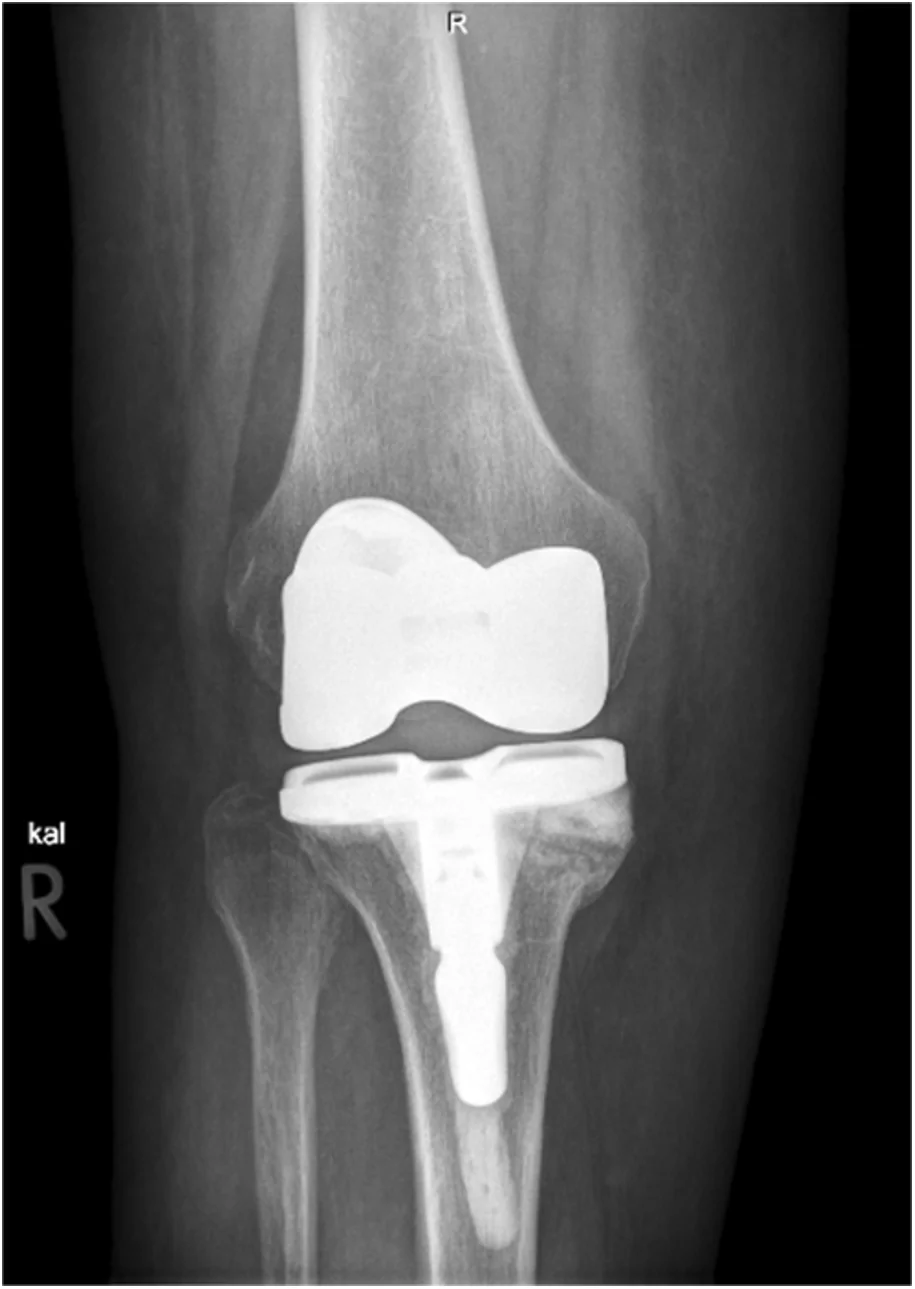
TKA with tibial stem after fractured UKA (a.p.). TKA, total knee arthroplasty; UKA, unicompartmental knee arthroplasty.

**Figure 7:**
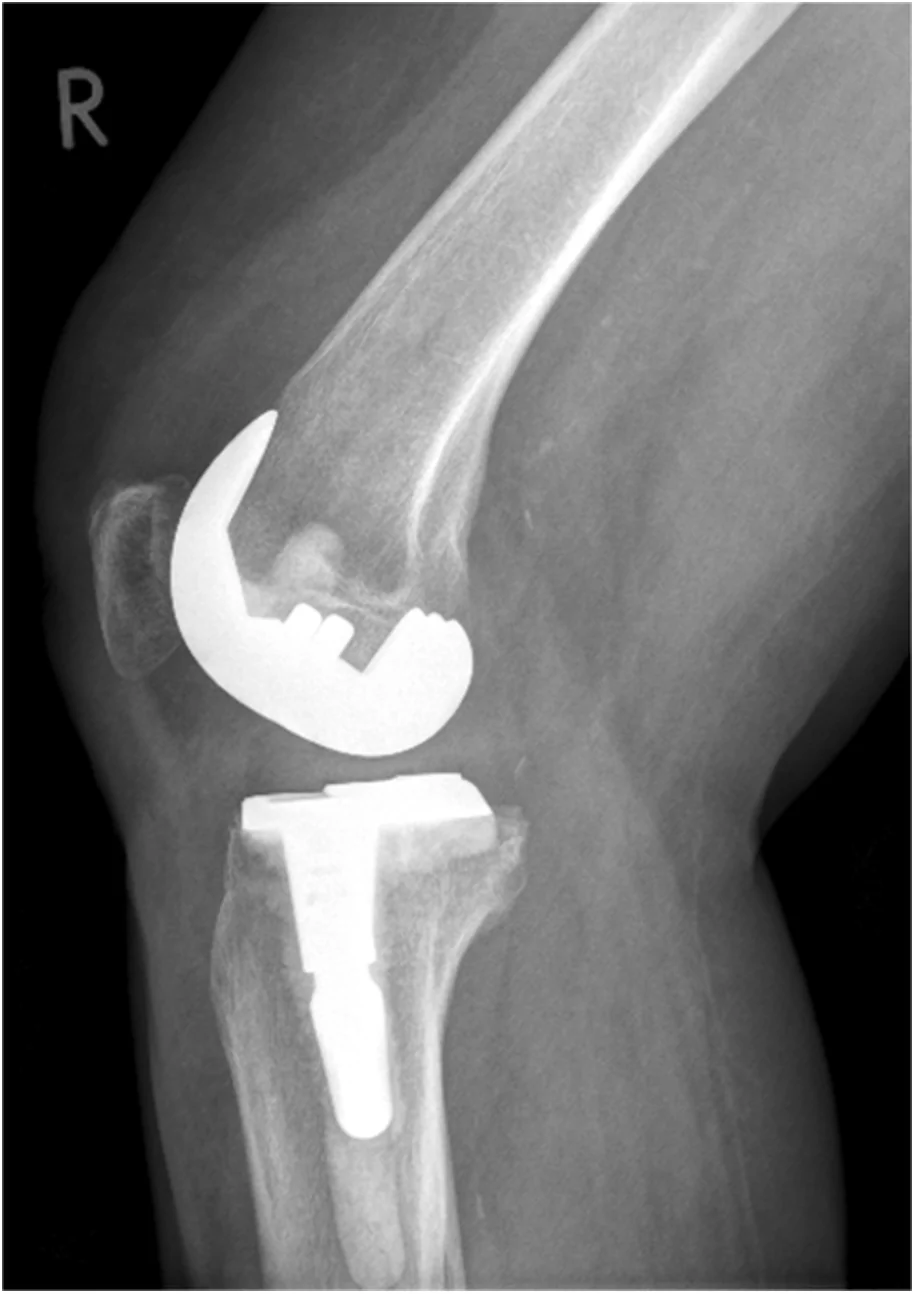
TKA with tibial stem after fractured UKA (lateral). TKA, total knee arthroplasty; UKA, unicompartmental knee arthroplasty.

PS components were used in 17 cases (63%), including 11 without and 6 with cemented tibial stem extensions (Figures 6 and 7). CPS components were used in 9 cases (33%), all with cemented tibial stem extensions. One patient (4%) received a LCCK prosthesis with cemented tibial stem extension.

Cemented tibial stems were used in 16 patients (59%), whereas 11 patients (41%) received no stem extension. All CPS and CCK implants were implanted with a stem, while only some PS implants were combined with a stem, based on intraoperative bone quality assessment. Implant types and the use of tibial stem extensions are presented in Table 3.

**Table 3:** Implant types and fixation.

Implant type	*n* (%)	With stem extension *n* (%)
Posterior‐stabilised (PS)	17 (63)	6 (35)
Constrained PS (CPS)	9 (33)	9 (100)
Constrained condylar knee (CCK)	1 (4)	1 (100)

### Re‐revisions and implant survival

At the latest follow‐up, 2 of 27 patients (7%) required re‐revision: one for tibial component loosening at 59 months and one for instability with femoral loosening at 47 months postconversion.

**Figure 8:**
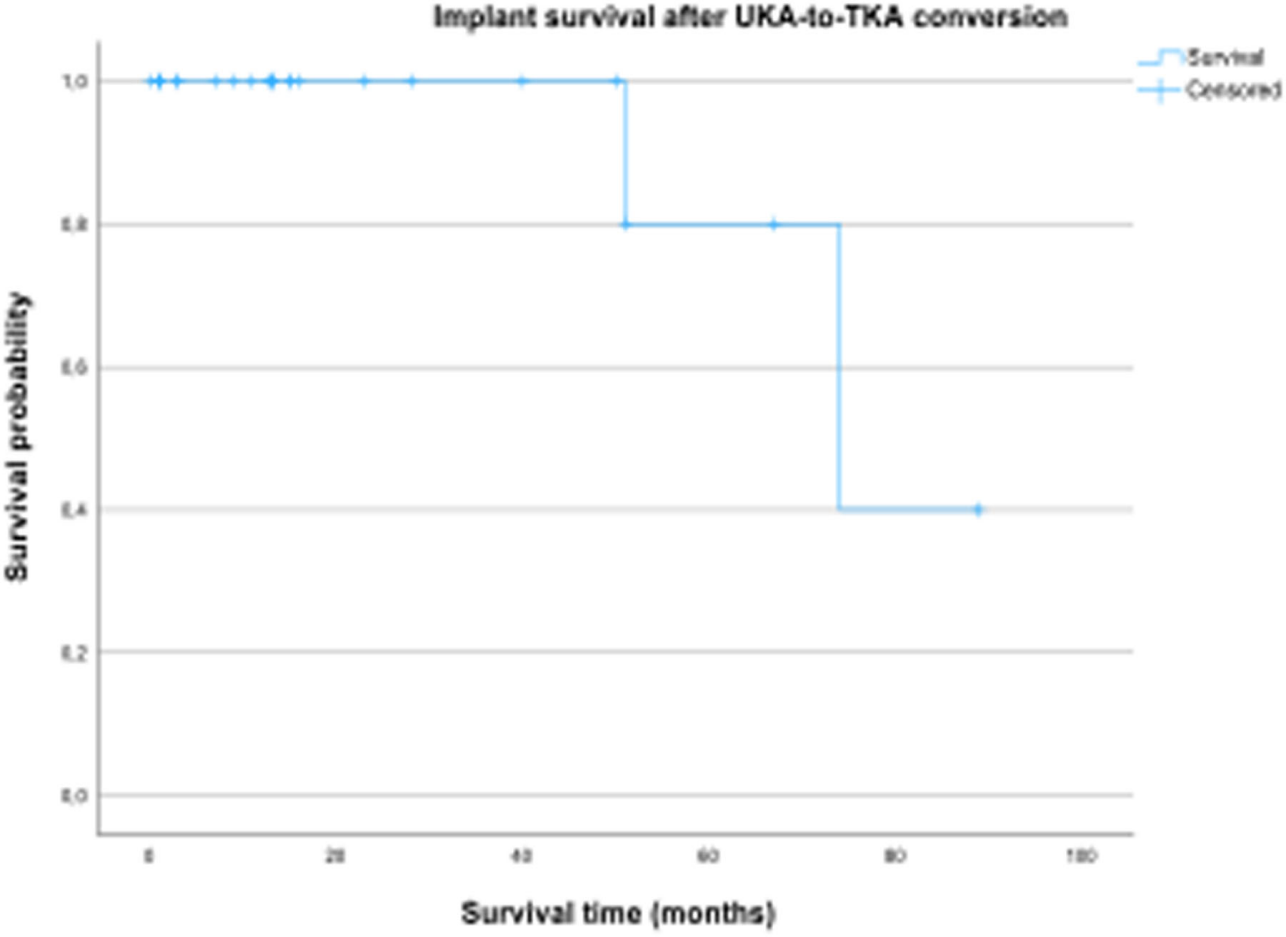
Implant survival after UKA‐to‐TKA conversion. TKA, total knee arthroplasty; UKA, unicompartmental knee arthroplasty.

Kaplan–Meier analysis estimated a median implant survival of 74 months (SD: 17.06; 95% CI: 40.57–107.43 months) following UKA‐to‐TKA conversion. The number of patients at risk at 12, 24, 36, 48 and 60 months was 27, 22, 18, 12 and 6, respectively (Figure 8).

Details of the re‐revisions performed after UKA‐to‐TKA conversion are shown in Table 4 and the implant survival is shown in Figure 8.

**Table 4:** Revisions after UKA‐to‐TKA.

Reasons for re‐revision	Time after conversion (months)	Implant type
Tibial component loosening (*n* = 1)	59	PS
Instability (*n* = 1)	47	PS

Abbreviations: PS, posterior‐stabilised; TKA, total knee arthroplasty; UKA, unicompartmental knee arthroplasty.

## DISCUSSION

The most important finding of this single‐centre series was that PS implants were used in most UKA‐to‐TKA conversions when intraoperative assessment indicated sufficient bone quality and knee stability. A minority required a higher constraint (CPS or CCK), and re‐revision was low. These observations are purely descriptive and reflect the practice in our high‐volume centre.

Moreover, periprosthetic tibial fracture emerged as the leading indication for conversion of UKA to TKA, followed by tibial component loosening and progression of osteoarthritis.

A recent systematic review on revision indications for medial UKA could show that aseptic loosening and OA progression are the two most common reasons for UKA failure [22]. Additionally, a multicentre study investigated that aseptic loosening—especially of the tibial component—emerge as the most frequent failure mode after UKA [7]. However, registry and contemporary review data also underscore that periprosthetic fracture, while uncommon overall, is a relevant and sometimes early complication in UKA patients [14, 20, 24]. An overview carried out by Mohr et al., as well as a systematic review and meta‐analysis performed by Burger et al., go along and could demonstrate that a majority of periprosthetic tibial fractures occur perioperatively or within the early phase after surgery [6, 15].

A recently published study, which also looked at UKA‐to‐TKA conversions, showed that the mean implant survival after 2 years is significantly higher for PS compared to rotating‐hinge designs (93% vs. 63%) [5].

Scheele et al. addressed this topic in a retrospective analysis, comparing patients who underwent primary UKA, those with primary TKA, those with UKA‐to‐TKA conversion, and those with revised TKA using various patient‐reported outcome measures (PROMs) to support clinical decision‐making [19]. One central finding of this study was that converted UKA are comparable to those with primary TKA, measured in terms of clinical outcomes and patient satisfaction [19]. While aiming for PS primary components to reach higher flexion [23], constrained options were reserved for defined indications such as severe ligamentous insufficiency and relevant bone loss, which follows recent findings [3].

However, other comparative analyses also show that relative to primary TKA, UKA conversions more often require stems, augments and thicker inserts to manage contained or uncontained defects and restore stability [11, 21].

Nevertheless, reinforcing the general principle that complex reconstruction options should remain readily available is important, since UKA‐to‐TKA conversion can be technically demanding [24]. These technical challenges of UKA revision can be joint exposure, component removal, bone defects and instability, as Vasso et al. claim [24]. Abdelaziz et al. conducted a retrospective review on aseptic revision TKA. Their investigation led them to claim that rotating‐hinge designs should be used to reduce stress shielding [1].

However, other series report higher complication or failure rates with hinged designs compared with less‐constrained implants [3, 5, 17], which supports a restrained, indication‐driven use rather than routine deployment in UKA‐to‐TKA conversions.

In our practice, this translated into selective use of constraint and routine stemmed fixation when a condylar‐constrained insert was chosen, consistent with contemporary recommendations.

Compared with primary TKA, patients revised from UKA tend to have inferior functional scores and range of motion and are more likely to require stems or augments, although short‐ to mid‐term revision rates may be similar, as Sun et. al. could demonstrate [21].

The implant survival within our cohort was satisfactory at midterm follow‐up, with few re‐revisions (*n* = 2) observed.

The comparably low re‐revision number and the observation that most reconstructions did not necessitate high degrees of constraint should be interpreted cautiously but may reflect institutional factors.

Roof et. al. conducted a retrospective study and could show that high‐volume surgeons reduced the re‐revision rate following revision‐TKA [18]. Moreover, Halder et al. found evidence that low‐volume hospitals have a significantly higher risk for revision surgery [9].

Taken together, these data offer a possible explanation for technically streamlined conversions in the hands of experienced surgeons in a high‐volume setting.

In summary, our data could serve as a notice that periprosthetic tibial fracture can be an important cause of UKA‐to‐TKA conversion, and that a high percentage of these conversions can be performed successfully with PS implants, supplemented selectively with stems or augments.

High surgeon volume and centre experience may appear linked to better survivorship and lower complication or re‐revision rates. These results support a surgical strategy that prioritises primary‐style reconstruction when feasible, while maintaining readiness for more complex reconstructive tactics.

This study has several limitations. The cohort was relatively small, which reduces the strength of statistical inferences. In addition, follow‐up duration varied and was short in several cases (Figure 1), resulting in only a limited number of re‐revision events. Consequently, the Kaplan–Meier curve should be viewed with some caution. As a retrospective single‐centre analysis, there is also a potential risk of selection bias. Furthermore, functional outcomes and PROMs were not available, restricting the analysis to implant survival and choice of constraint. Finally, the absence of a direct comparison group, such as primary TKA or UKA‐to‐TKA conversions from other centres, limits the generalisability of the results. Despite these limitations, the study benefits from a homogeneous patient cohort, a consistent surgical workflow, and procedures performed by experienced high‐volume surgeons, which supports the internal validity of these observations.

## CONCLUSION

While conversion of medial UKA to TKA is often associated with complex implant use and elevated re‐revision rates, our findings suggest that in a specialised high‐volume centre with a consistent surgical workflow, most conversions could be performed with PS TKA implants and re‐revisions remain rare.

Under these conditions, the conversion procedure may more closely resemble a primary TKA than a complex revision case in terms of the choice of implants.

## AUTHOR CONTRIBUTIONS

**Julius Brendler:** Data collection; introduction; methodology; analysis; results; discussion; conclusion. **Lorenzo Impieri:** Introduction, data collection. **Christoph Linhart:** Conceptualisation and review. **Michael Najfeld:** Conceptualisation and review. **Robert Hube:** validation and review. **Daniel Pfeufer:** Conceptualisation; validation; editing and review. All authors have read and agreed to the published version of the manuscript.

## CONFLICT OF INTEREST STATEMENT

All authors declare no conflicts of interest related to this work, except for Robert Hube, who reports receiving financial benefits from Zimmer Biomet®.

## ETHICS STATEMENT

The study was conducted in accordance with the Declaration of Helsinki and approved by the local ethics committee (Ethik‐Kommission der BLÄK, Mühlbauerstraße 16, 81677 München, approval no. 24075). Since routine patient data was retrospectively analysed, no informed consent was necessary.

## Data Availability

The data that support the findings of this study are available from the corresponding author upon reasonable request.
